# Machine-Learning-Driven Reconstruction of Organic Aerosol Sources across Dense Monitoring Networks in Europe

**DOI:** 10.1021/acs.estlett.5c00771

**Published:** 2025-10-20

**Authors:** Adrien Jouanny, Abhishek Upadhyay, Jianhui Jiang, Petros Vasilakos, Marta Via, Yun Cheng, Benjamin Flueckiger, Gaëlle Uzu, Jean-Luc Jaffrezo, Céline Voiron, Olivier Favez, Hasna Chebaicheb, Aude Bourin, Anna Font, Véronique Riffault, Evelyn Freney, Nicolas Marchand, Benjamin Chazeau, Sébastien Conil, Jean-Eudes Petit, Jesús D. de la Rosa, Ana Sanchez de la Campa, Daniel Sanchez-Rodas Navarro, Sonia Castillo, Andrés Alastuey, Xavier Querol, Cristina Reche, María Cruz Minguillón, Marek Maasikmets, Hannes Keernik, Fabio Giardi, Cristina Colombi, Eleonora Cuccia, Stefania Gilardoni, Matteo Rinaldi, Marco Paglione, Vanes Poluzzi, Dario Massabò, Claudio Belis, Stuart Grange, Christoph Hueglin, Francesco Canonaco, Anna Tobler, Hilkka J. Timonen, Minna Aurela, Mikael Ehn, Iasonas Stavroulas, Aikaterini Bougiatioti, Konstantinos Eleftheriadis, Maria I. Gini, Olga Zografou, Manousos-Ioannis Manousakas, Gang Ian Chen, David Christopher Green, Petra Pokorná, Petr Vodička, Radek Lhotka, Jaroslav Schwarz, Andrea Schemmel, Samira Atabakhsh, Hartmut Herrmann, Laurent Poulain, Harald Flentje, Liine Heikkinen, Varun Kumar, Hugo Anne Denier van der Gon, Wenche Aas, Stephen M. Platt, Karl Espen Yttri, Imre Salma, Anikó Vasanits, Benjamin Bergmans, Yulia Sosedova, Jaroslaw Necki, Jurgita Ovadnevaite, Chunshui Lin, Julija Pauraite, Michael Pikridas, Jean Sciare, Jeni Vasilescu, Livio Belegante, Célia Alves, Jay G. Slowik, Nicole Probst-Hensch, Danielle Vienneau, André S. H. Prévôt, Aniss Aiman Medbouhi, Daniel Trejo Banos, Kees de Hoogh, Kaspar R. Daellenbach, Ekaterina Krymova, Imad El Haddad

**Affiliations:** † PSI Center for Energy and Environmental Sciences, 5232 Villigen, Switzerland; ‡ Global Institute for Urban and Regional Sustainability, School of Ecological and Environmental Sciences, 12655 East China Normal University, Shanghai 200231, China; § Center for Atmospheric Research, 119110 University of Nova Gorica, SI-5000 Nova Gorica, Slovenia; ∥ Swiss Data Science Center, 685401 EPFL and ETH Zürich, 8092 Zürich, Switzerland; ⊥ 30247 Swiss Tropical and Public Health Institute, Kreuzstraße 2, 4123 Allschwil, Switzerland; # 27015 University Grenoble Alpes, CNRS, IRD, INP-G, INRAE, IGE (UMR 5001), 38000 Grenoble, France; 7 52805 Institut National de l’Environnement Industriel et des Risques (INERIS), 60550 Verneuil-en-Halatte, France; 8 505883 IMT Nord Europe, Institut Mines-Télécom, Université de Lille, Centre for Energy and Environment, 59000 Lille, France; 9 Laboratoire de Météorologie Physique, 124097 Université Clermont Auvergne−CNRS, 63170 Aubière, France; 10 Aix Marseille University, CNRS, LCE, 13003 Marseille, France; 11 124095 Agence Nationale pour la Gestion des Déchets Radioactifs (ANDRA), RD960, 55290 Bure, France; 12 113461 Laboratoire des Sciences du Climat et de l’Environnement (LSCE), 91190 Gif-sur-Yvette, France; 13 Center for Research in Sustainable Chemistry (CIQSO), Associate Unit CSIC−University of Huelva “Atmospheric Pollution”, Campus El Carmen s/n, 21071 Huelva, Spain; 14 Department of Applied Physics, 117396 Universidad de Granada, Av. de la Fuente Nueva, 18071, Granada, Spain; 15 Institute of Environmental Assessment and Water Research (IDAEA-CSIC), 08034 Barcelona, Spain; 16 Air Quality and Climate Department, Estonian Environmental Research Centre (EERC), Marja 4D, 10617 Tallinn, Estonia; 17 Institute of Physics, 226264 University of Tartu, 50411 Tartu, Estonia; 18 479462 National Institute for Nuclear Physics−Florence Section, Via Sansone 1, 50019 Sesto Fiorentino, Florence, Italy; 19 Environmental Protection Agency of Lombardy (ARPA Lombardia), 20124 Milan, Italy; 20 Italian National Research Council, Institute of Polar Sciences (CNR−ISP), 20125 Milan, Italy; 21 Italian National Research Council−Institute of Atmospheric Sciences and Climate (CNR−ISAC), 40129 Bologna, Italy; 22 Arpae Emilia-Romagna, Centro Tematico Regionale Qualità dell’Aria, 40139 Bologna, Italy; 23 Department of Physics, 276249 University of Genoa & INFN-Genoa, 16146 Liguria, Italy; 24 99013 European Commission, Joint Research Centre, 21027 Ispra, Italy; 25 School of Earth and Atmospheric Sciences, 124388 Queensland University of Technology, Gardens Point, Brisbane, Queensland 4000, Australia; 26 28501 Empa, Swiss Federal Laboratories for Materials Science and Technology, 8600 Dübendorf, Switzerland; 27 Datalystica, Limited, 5234 Villigen, Switzerland; 28 Atmospheric Composition Research, 52931 Finnish Meteorological Institute, 00560 Helsinki, Finland; 28a Aerosol Physics Laboratory, Tampere University, 33100 Tampere, Finland; 29 Institute for Atmospheric and Earth System Research/Physics, Faculty of Science, University of Helsinki, 00014 Helsinki, Finland; 30 Institute for Environmental Research and Sustainable Development, 54571 National Observatory of Athens, 15236 Athens, Greece; 31 Environmental Radioactivity & Aerosol Technology for Atmospheric & Climate Impact Lab, INRaSTES, NCSR “Demokritos”, 15310 Athens, Greece; 32 MRC Centre for Environment and Health, Environmental Research Group, 4615 Imperial College London, 86 Wood Lane, London W12 0BZ, United Kingdom; 33 HPRU in Environmental Exposures and Health, Imperial College London, 86 Wood Lane, London W12 0BZ, United Kingdom; 34 Institute of Chemical Process Fundamentals, Czech Academy of Sciences, 165 00 Prague 6, Czech Republic; 35 39417 Umweltbundesamt, 06844 Dessau-Roßlau, Germany; 36 28397 Leibniz Institute for Tropospheric Research (TROPOS), 04318 Leipzig, Germany; 37 64386 German Meteorological Service (DWD), 82383 Hohenpeissenberg, Germany; 38 Department of Environmental Science, 7675 Stockholm University, 114 18 Stockholm, Sweden; 38a Bolin Centre for Climate Research, Stockholm University, 114 18 Stockholm, Sweden; 39 Department of Environmental Sciences, 307855 Aarhus University, DK-4000 Aarhus, Denmark; 40 Department of Climate, Air and Sustainability, 2859 TNO, 3584 CB Utrecht, Netherlands; 41 87482 NILU, 2007 Kjeller, Norway; 42 Institute of Chemistry, 54616 Eötvös Loránd University, 1053 Budapest, Hungary; 43 Institut Scientifique de Service Public (ISSEP), 4000 Liège, Belgium; 44 Faculty of Physics and Applied Computer Science, 514448 AGH University of Krakow, 30-059 Krakow, Poland; 45 School of Natural Sciences, Physics, Centre for Climate and Air Pollution Studies, Ryan Institute, 8799 University of Galway, Galway H91 CF50, Ireland; 46 State Key Laboratory of Loess Sciences, Center for Excellence in Quaternary Science and Global Change, Institute of Earth Environment, 53047 Chinese Academy of Sciences, Xi’an 710061, China; 47 SRI Center for Physical Sciences and Technology (FTMC), 10257 Vilnius, Lithuania; 48 Climate and Atmosphere Research Center (CARE-C), The Cyprus Institute, 2121 Nicosia, Cyprus; 49 124257 National Institute of Research and Development for Optoelectronics (INOE 2000), 077125 Magurele, Romania; 50 Department of Environment and Planning, Centre for Environmental and Marine Studies (CESAM), 56062 University of Aveiro, 3810-193 Aveiro, Portugal; 51 University of Basel, 4001 Basel, Switzerland; 52 Department of Intelligent Systems, 641674 KTH Royal Institute of Technology, 11428 Stockholm, Sweden; 53 College of Environmental Sciences and Engineering, Peking University, Beijing 100084, China

**Keywords:** source apportionment, machine learning, deep learning, Europe data set, spatial−temporal analysis, air quality, organic aerosols

## Abstract

Fine particulate matter (PM) poses a major threat to public health, with organic aerosol (OA) being a key component. Major OA sources, hydrocarbon-like OA (HOA), biomass burning OA (BBOA), and oxygenated OA (OOA), have distinct health and environmental impacts. However, OA source apportionment via positive matrix factorization (PMF) applied to aerosol mass spectrometry (AMS) or aerosol chemical speciation monitoring (ACSM) data is costly and limited to a few supersites, leaving over 80% of OA data uncategorized in global monitoring networks. To address this gap, we trained machine learning models to predict HOA, BBOA, and OOA using limited OA source apportionment data and widely available organic carbon (OC) measurements across Europe (2010–2019). Our best performing model expanded the OA source data set 4-fold, yielding 85 000 daily apportionment values across 180 sites. Results show that HOA and BBOA peak in winter, particularly in urban areas, while OOA, consistently the dominant fraction, is more regionally distributed with less seasonal variability. This study provides a significantly expanded OA source data set, enabling better identification of pollution hotspots and supporting high-resolution exposure assessments.

## Graphical abstract

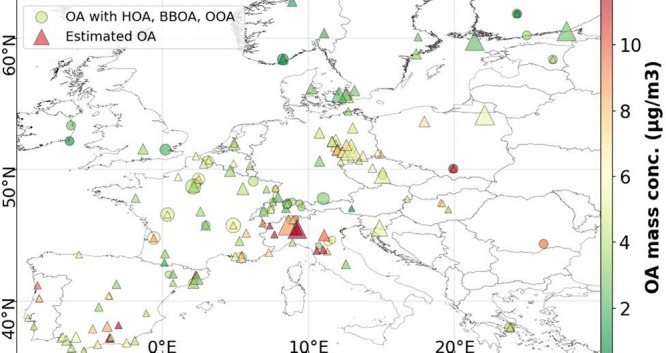

## 1 Introduction

The World Health Organization (WHO) recently updated its air quality guidelines, setting stricter limits for fine particulate matter (PM_2.5_) concentrations at 5 μg m^–3^ to mitigate PM health impacts.^1^ Currently, over 97% of the European population lives in areas exceeding these limits.^2^ Mitigating PM mass concentrations requires high-resolution temporal and spatial data on its chemical composition and sources.

Organic aerosol (OA) is the most chemically complex PM fraction, consisting of millions of individual compounds. It can originate from local primary sources, such as mainly traffic emissions and industry (hydrocarbon-like OA (HOA)] or residential biomass burning [biomass burning OA (BBOA)]. It can also form in the atmosphere through the condensation of oxidation products of volatile organic compounds, known as secondary OA (SOA) or oxygenated OA (OOA). Currently, OA accounts for over half of PM mass in Europe and is expected to play an even larger role as regulatory measures reduce SO_2_ and NO_*x*_ emissions as precursors of secondary inorganic compounds.^3^ This shift is compounded by climate change, which likely increases natural emissions of OA precursors.^4^

OA can be measured online using an aerosol mass spectrometer (AMS) and aerosol chemical speciation monitor (ACSM).^5,6^ Using positive matrix factorization (PMF), these measurements provide the apportionment of OA fractions based on their spectral fingerprints, including HOA, BBOA, and OOA^3^ (section S.1). However, OA measurements and source apportionment are resource-intensive, requiring significant financial investment and skilled personnel. As a result, AMS and ACSM deployments remain limited to a few locations, with long-term ACSM measurements available primarily at supersite networks, such as ACTRIS^7^ in Europe, ASCENT^8^ in the U.S., and selected sites in China.^9^ In contrast, total OA is more widely monitored as organic carbon (OC), which is measured offline on filter samples at larger networks, such as EMEP^10^ in Europe and IMPROVE^11^ in the U.S. While these networks may provide broader spatial and temporal coverage, OC measurements lack source information.

Here, we developed a machine learning (ML) approach to integrate OA source apportionment data from 44 stations (Table S1) with widespread OC measurements from 136 stations (Table S2) across Europe. This method expanded the OA source apportionment data set 4-fold, enabling the estimation of the contributions from three main OA components, HOA, BBOA, and OOA, resulting in a total of ∼85 000 daily concentration values between 2010 and 2019 at 180 stations.

## 2 Materials and Methods

We model the contribution of three source components, HOA, BBOA, and OOA, using tree ensembles and deep neural networks (DNNs). The modeling framework is shown in Figure 1. ML models filled in source-specific information at locations where only total OC data exists, incorporating inputs from chemical transport model (CTM) outputs, land-use data, weather conditions, and total measured OC concentrations. Chemical transport models provided information into emission sources, atmospheric dynamics, and reactivity, while high-resolution land-use data capture factors for local variability. Models were trained using the source-specific OA database, and model performance was evaluated using the leave-one-station-out (LOSO) method. Model inputs are detailed in section 2.1 and are presented in Table S3. The implementation of tree-based and deep-learning models is described in section 2.2, and the model description and parameters are summarized in Table S4.

**Figure 1:**
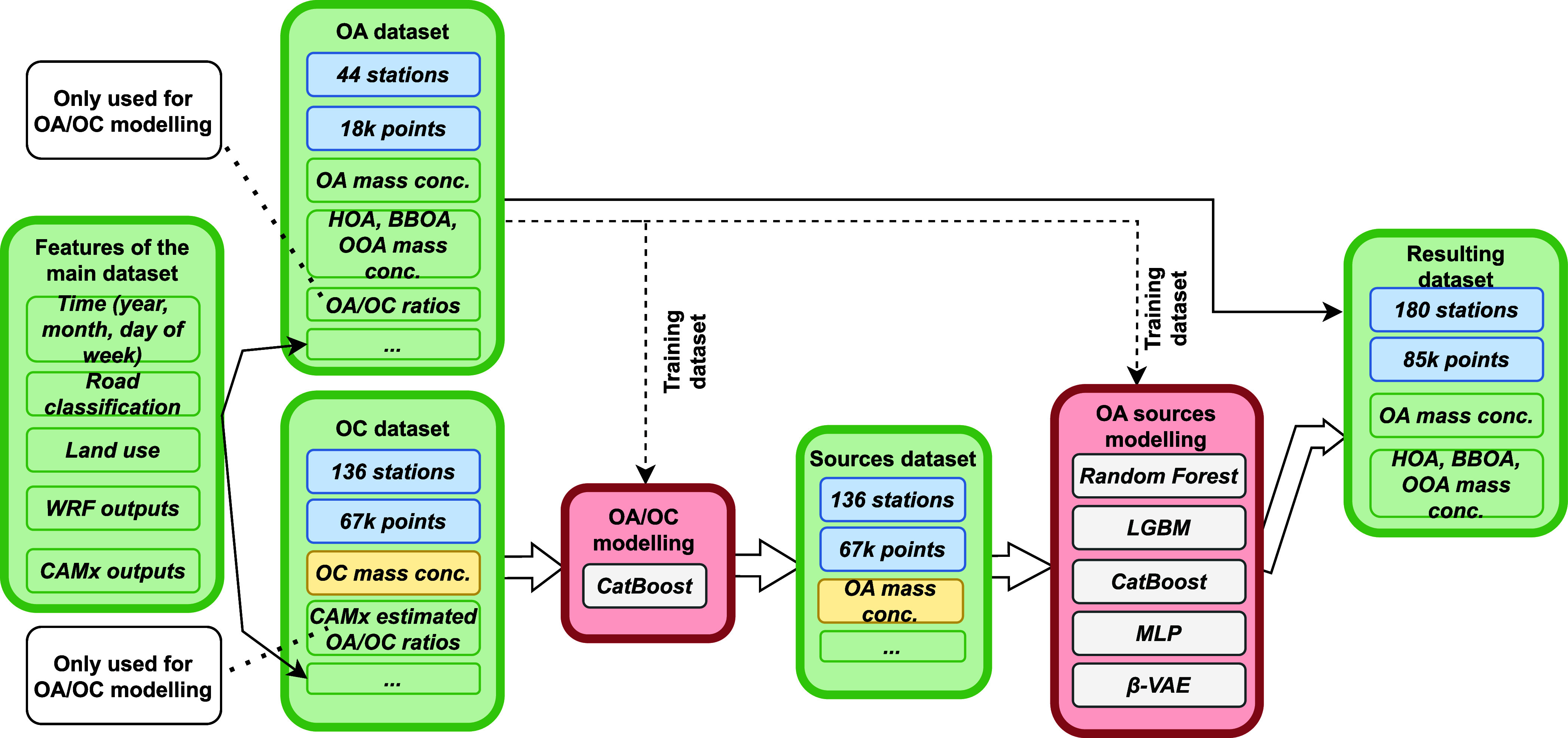
This workflow diagram illustrates the integration of two data sets (one with OA and its sources and one with OC) to model and predict OA sources using OC. The OA and OC data sets, derived from monitoring stations, are enriched with temporal, land-use, meteorological, and chemical transport model outputs. A CatBoost model is applied to estimate OA/OC ratios on the OC data set. Then, several machine learning methods, such as random forest, LGBM, CatBoost, MLP, and β-VAE, are used to apportion the estimated OA. This process results in the creation of a data set with 85 000 HOA, BBOA, and OOA estimations across 180 monitoring stations.

### 2.1 Model Inputs

#### OC and OA Component Data

OA source apportionment data, resulting from the application of PMF to ACSM and AMS measurements, are available at 44 European sites,^3^ comprising a total of 18 000 daily values from 2013 to 2019. Measurements and PMF methodology for the ACSM data set are described in the data reference for each data set (Table S1/section S.1). We focused on three consistently apportioned components, HOA, BBOA, and OOA, while excluding locally specific sources, such as cooking emissions, identified at only a few sites. In some locations, two OOA components were resolved from PMF, which we combined into a single total OOA value to keep three consistent components for all of the sites. Total OC, without a source apportionment, is measured using a thermal–optical technique at 136 European sites, totaling 67 000 daily values from 2010 to 2019. A detailed temporal distribution of sampling frequency and site coverage, including annual and monthly data, is presented in Figure S1 of the Supporting Information. Since we have two separate data sets, one for OC and another for OA along with the OA/OC ratio, we required a dedicated model to estimate OA from OC. While CTM outputs (see below) provide OA/OC estimates (Table S5), we sought to enhance accuracy using a gradient boosting method, CatBoost.^12^ We used CTM outputs, land-use data, and meteorological variables as predictors (Table S5). The model was trained on OA/OC ratios from AMS/ACSM data derived from chemical composition (Table S5). Finally, the model was applied to calculate the OA/OC ratios for all offline samples to deduce OA mass concentrations.

We noted that AMS/ACSM data sets correspond mostly to the PM_1_ fraction where the overwhelming majority of OA, HOA, BBOA, and OOA mass occurs, while OC is measured using either PM_2.5_ and PM_10_ samples. Approximately 80–90% of the HOA, BBOA, and OOA mass typically falls within the PM_1_ fraction of the PM_2.5_ sample.^13^ Therefore, we expect to slightly overestimate the contributions of OA, HOA, BBOA, and OOA when using OA estimated from OC measurements due to small contributions from biological aerosols in the coarse mode.

#### CTM Outputs

We used the Comprehensive Air Quality Model with Extensions^14^ (CAMx, version 6.5) to simulate daily organic aerosol (OA) source components, inorganic aerosols, trace gases, and oxidant concentrations across Europe for the period 2010–2019. The simulation domain covered Europe with a spatial resolution of 0.25° × 0.125°. Meteorological data from Weather Research and Forecasting^15^ (WRF) model simulations were used in offline mode. CAMx used annual anthropogenic emissions from the European emission inventory Copernicus Atmosphere Monitoring Service–Regional inventory, version 4 (CAMS-REG-v4), developed by TNO.^16^ PM emissions are split into various chemical components, including OC, using PM splitting factors specific to source and country for fine and coarse aerosol modes from TNO. We used monthly, weekly, and hourly temporal splitting profiles from TNO to generate hourly emissions. Biogenic volatile organic compounds (BVOCs) from plants, critical precursors for secondary organic aerosols (SOAs), were generated using the PSI model.^17,18^ Transported wildfire BBOA emissions were not included in CAMx simulations, as their influence on European air quality is generally minor and episodic. Outputs from the CAMx simulations used as inputs for ML models are detailed in Tables S3 and S5.

#### Land-Use Data

Land-use variables, harmonized to a 200 m resolution and an annual scale from their native resolution for the entire Europe, were used as ML model inputs (Table S3 and S5), including the fractions of 14 land-use types (e.g., urban fraction, natural green, agriculture, and barren land), population density, altitude, and road length and category.

#### Weather Data

Daily means of the temperature, surface pressure, wind speed and direction, relative humidity, and planetary boundary layer height simulated using the Weather Research and Forecasting model (WRF, version 4.1) are used as inputs for the ML models (Table S3 and S5).

### 2.2 ML Models

We have implemented and optimized the following tree-based models: random forest (RF)^19^ from scikit-learn,^20^ gradient boosting from CatBoost,^12^ and LGBM.^21^ Targets, HOA, BBOA, and OOA, were scaled using a ln­(1 + *y*_target_) transformation, a common technique used for unskewing the data and making it closer to normally distributed.

Two DNNs have been employed: multilayer perceptron^22,23^ (MLP) and variational autoencoders^24^ (VAEs). Such models allow us to easily implement a sum constraint between targets. Specifically, we enforced the following constraint using a SoftMax layer:^25,26^ $1=\frac{\mathrm{HOA}}{\mathrm{OA}}+\frac{\mathrm{BBOA}}{\mathrm{OA}}+\frac{\mathrm{OOA}}{\mathrm{OA}}+\frac{\mathrm{residuals}}{\mathrm{OA}}$ (residuals represent locally specific sources). This approach led to improved prediction accuracy, particularly for HOA and BBOA. DNNs are prone to overfitting, as noise can significantly impact training.^25^ To mitigate this issue, we implemented several regularization techniques, including batch normalization,^27^ dropout,^28^ and early stopping^29^ in the MLP. We also explored a β-VAE^30^ approach, which balances reconstruction accuracy with a well-structured latent space. By applying pseudo-Gibbs sampling to the β-VAE predictions, we generated multiple estimates for the missing OA components, thereby capturing their uncertainty.^31^

## 3 Results and Discussion

### 3.1 OC to OA Conversion and OA Distribution

We estimated the OA/OC ratio using the method described in section 2.1 and evaluated the performance using LOSO cross-validation. This approach outperformed OA/OC ratios derived from CTM, achieving a root-mean-square error (RMSE) in OA/OC of 0.055 (i.e., <5%) compared to a CAMx RMSE of 0.123 (i.e., ∼10%). Figure S2 illustrates the temporal trend of the OA/OC ratio for both rural and urban stations. The minimum OA/OC ratio averaged per station was 1.64 in Athens (Greece), while the highest was 1.81 in Campisabalos (Spain).

Using OA/OC ratios, we deduced the OA mass concentration across Europe (from 2010 to 2019) shown on Figure 1. This extended data set contains an average of 478 OA daily concentrations per site. The highest OA concentrations occurred in the vicinity of Florence, Italy (19.0 μg m^–3^), while the lowest levels were found at the Zeppelin Mountain, Svalbard (0.2 μg m^–3^). The spatial distribution of sampling sites revealed greater station density in western and southwestern Europe compared to those in northern and eastern regions.

### 3.2 Model Performance

Model performance was evaluated using LOSO cross-validation, 44-fold covering all stations where the OA component is available. In this approach, the model was trained on data excluding a specific station and then tested on that station to assess its predictive accuracy. Performance was quantified using the coefficient of determination (*R* ^2^), RMSE, and mean absolute percentage error (MAPE) metrics. Figure S3 illustrates the distribution of *R* ^2^ scores achieved by the five different models.

Figure S3 demonstrates that all models achieve comparable performance in terms of median *R* ^2^. All models predict the level of OOA with high accuracy, achieving a median *R* ^2^ of approximately 0.8, as the level of OOA represents the largest fraction of OA. In contrast, HOA and BBOA, which contribute less to OA, were more challenging to predict, with median *R* ^2^ values of around 0.4 and 0.6, respectively.

Among the models, CatBoost consistently outperformed the others, exhibiting higher 10th and 25th *R* ^2^ percentiles, which signifies a robust performance across most stations. In comparison, RF, which employs a simple ensemble strategy by averaging independent trees, struggled to capture observed patterns uniformly across all stations.^19^ Similarly, LGBM showed a slight underfitting, probably because of its histogram-based decision tree learning approach.^21^ Both the RF and LGBM models are inherently constrained in extrapolating beyond the training data, leading to bounded predictions. DL models generally perform on par with the tree-based models for most stations, showing no significant advantages or drawbacks. Figure S4 shows HOA, BBOA, and OOA time series for the Lille (France) station using the six different models and CAMx, illustrating that, for most of the stations, all five ML models capture the variation and the magnitude of OA components.

Given the importance of achieving consistent predictive accuracy across all stations, we selected the CatBoost model as the optimal choice. Figure S5 shows feature importance for the CatBoost model, which describes the model’s dependency on different input features; OA is the most important predictor for all components HOA, BBOA, and OOA. CAMx_BBOA and CAMx_OOA are key features for BBOA and OOA, respectively. For HOA, CAMx_EC and CAMx_BBOA are important, with EC reflecting similar traffic-related sources. In comparison to the CAMx, which yielded the following coefficients of determination $\{\begin{array}{l} R_{\mathrm{HOA}}^{2}=-0.17\\ R_{\mathrm{BBOA}}^{2}=-0.03\\ R_{\mathrm{OOA}}^{2}=-0.14 \end{array}$ , CatBoost achieved $\{\begin{array}{l} R_{\mathrm{HOA}}^{2}=0.60\\ R_{\mathrm{BBOA}}^{2}=0.58\\ R_{\mathrm{OOA}}^{2}=0.75 \end{array}$ . Figure 2 and Figure S6 show that the model slightly underestimated high values across all components but generally well-reproduced HOA, BBOA, and OOA estimated by PMF.

**Figure 2:**
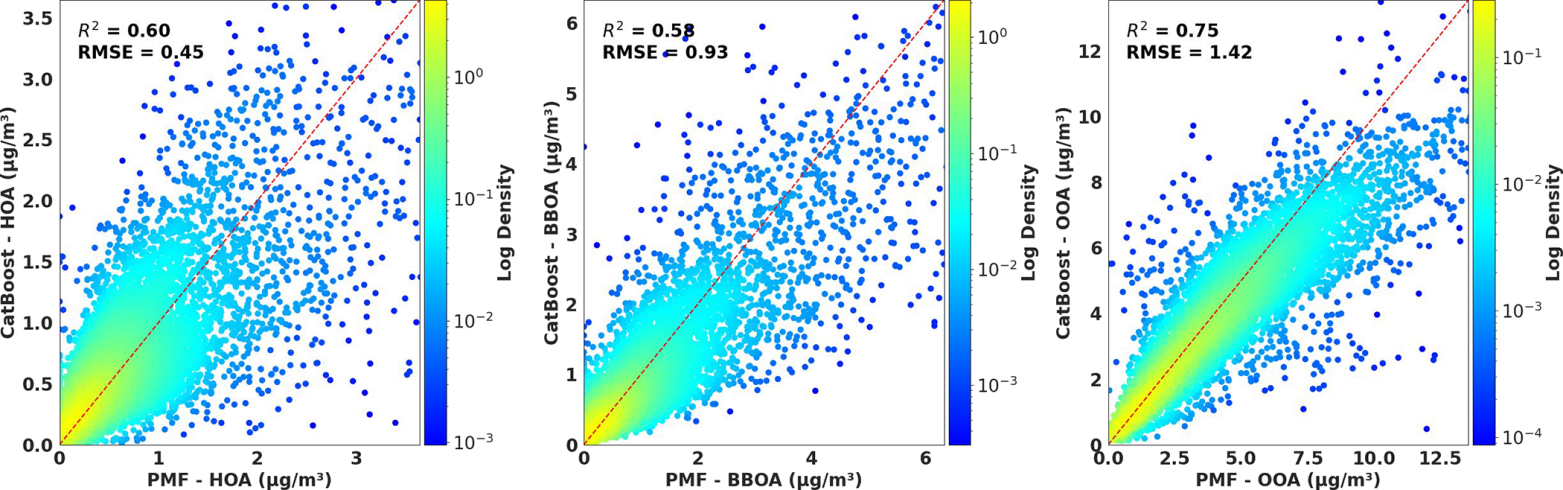
Scatter plots for HOA, BBOA, and OOA using the CatBoost model with log-transformed targets, points colored according to the log density of the best two-dimensional Gaussian. The red line corresponds to *y* = *x*. Figure S7 presents the same plots but for the CAMx model. Figure S11 presents the same plots but without using OA as an input (it significantly decreases the performance).

Analyzing feature importance using the CatBoost model (Figure S5) revealed that CAMx outputs strongly informed the prediction of BBOA and OOA but contribute less to HOA, highlighting challenges in accurately representing local HOA emissions with CAMx. Additionally, other CAMx outputs (e.g., NO_2_, elemental carbon, and CO), meteorological parameters (pressure and temperature), population density, and road length as well as temporal data (year/month) also played important roles.

The larger prediction errors observed for BBOA and HOA, relative to those of the OOA, are consistent with the generally higher uncertainties associated with PMF source apportionment of primary organic aerosol fractions. As formal PMF error analysis becomes more common, future studies should evaluate how these uncertainties propagate into supervised learning models and how they affect their predictive reliability.

### 3.3 Spatial and Temporal Variability of HOA, BBOA, and OOA

Using CatBoost, we apportioned OA components at 136 sites in Europe with OC data. This enables us to obtain a more robust representation of the spatial and monthly variability of OA components across Europe from 180 sites, over 4 times the monitored sites (Figure 3). The mean concentrations of HOA, BBOA, and OOA are 0.5 ± 0.3, 0.7 ± 0.7, and 3.2 ± 1.4 μg m^–3^ in Europe (mean ± standard deviation).

**Figure 3:**
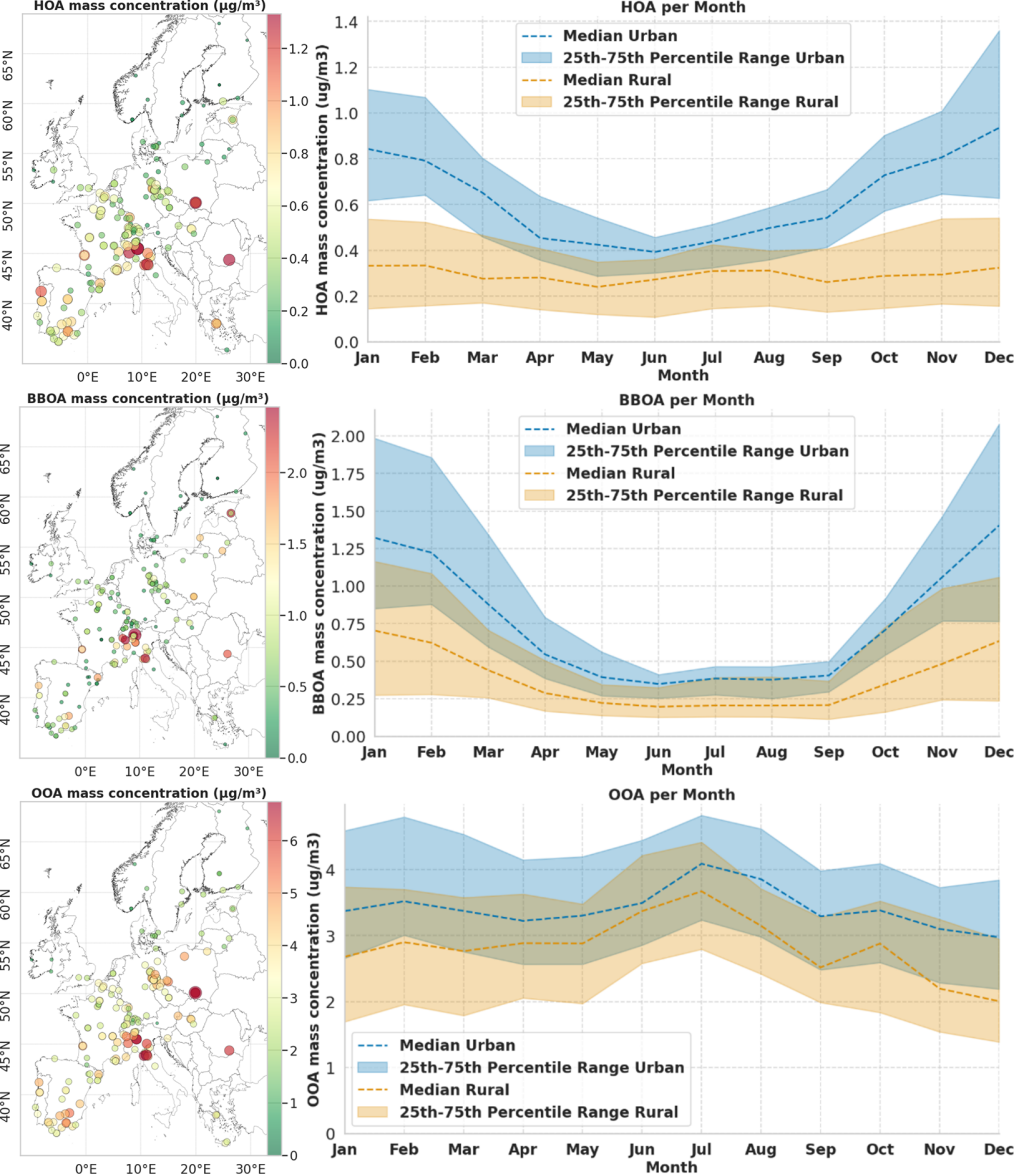
Spatial and temporal distributions of OA fractions. (Left) Europe maps with scatter dots showing the mean concentration of HOA, BBOA, and OOA at 180 locations across Europe. (Right) Line graph showing monthly trends in OA component concentrations from 180 locations. We differentiated urban and rural areas using land-use variables that correspond to population density and impervious surface density (IMD), as described in Table S3. Tables S1 and S2 show which stations are considered rural/urban. Figure S8 presents the same plots for the CAMx model. The size of the point is proportional with the OA mass concentration. The data link is provided in section S.2.

The highest concentrations of HOA, BBOA, and OOA were predicted in northern Italy (Po Valley and western Tuscany), Krakow (Poland), and Bucharest (Romania). The geography of the Po Valley causes air stagnation, allowing pollutants from urban and industrial areas to accumulate.^32^ Elevated HOA levels were observed in major western European cities, such as Paris, Lisbon, Madrid, and Barcelona, as well as in eastern cities, such as Bucharest and Krakow, driven primarily by urban traffic emissions. High BBOA concentrations were detected in rural areas near the Alps and in cities, such as Tartu, Vilnius, and Bucharest, reflecting emissions from residential heating. OOA showed significantly less spatial variability compared to HOA and BBOA, consistent with its regional formation process.

Seasonally, HOA and BBOA levels were higher in the winter, driven by increased heating and a shallower boundary layer, with BBOA showing a more pronounced increase. Winter increases in HOA and BBOA were more pronounced in urban areas, where road traffic emissions, heating demand, and population density are higher. In contrast, despite greater atmospheric dilution in the summer, the extent of adsorption of OOA shows a discrete seasonal variability, with a small peak observed between July and August, reflecting enhanced photochemical reactions and biogenic emissions. However, in comparison, CAMx consistently underestimates the concentrations of OOA during winter (Figure S8), likely due to missing anthropogenic precursor emissions or missing chemical processes (e.g., aqueous and particle phase chemistry). While urban areas consistently exhibited higher HOA and BBOA levels than rural areas, OOA levels were similar in urban and rural regions. The model shows spatial and seasonal patterns, highlighting the local influences of HOA and BBOA and the regional nature of OOA.

Although OA observations are limited in regions like southern Spain, Portugal, the Leipzig region (Germany), and Denmark, ample OC data allow our model to provide high-quality OA estimates there. By contrast, Eastern Europe has a few supersites but insufficient OC coverage, restricting the model’s utility, and the Balkans lack both OA and OC measurements entirely. Overall, the expanded OA data set supports diurnal and weekly analyses, chemical transport model evaluation, downscaling, site-specific causal inference, and policy development.

## 4 Implications

We have trained a ML model that integrates sparse OA source apportionment data, available only at supersites, with abundant OC measurements to estimate the major OA fractions, HOA, BBOA, and OOA, across large-scale networks in Europe. This method quadrupled data coverage, revealing spatial and seasonal patterns, and delivered a comprehensive data set of 85 000 daily concentrations of OA components from 180 sites. This data set can be used to infer the causal factors driving OA pollution in Europe and for training exposure models for future epidemiological analysis. Furthermore, we introduced a novel method for splitting OA into its major components, a common issue across measurement networks in the EU (ACTRIS), U.S. (IMPROVE and ASCENT), and China, where 80% of OA data remains uncategorized.

## Supplementary Material



## Notes

List of the 44 stations that have OA, HOA, BBOA, and OOA measurements, with the available number of daily concentrations (*n*), time range for data availability, and references describing measurement and PMF methodology (Table S1), 136 stations that have OC measurements, with *n* being the number of daily concentrations (Table S2), variables used as input features for HOA, BBOA, and OOA modeling (Table S3), description of used machine learning and deep learning models (Table S4), variables used as features in OA/OC ratio modeling (Table S5), distribution of the number of sites per year and per month with OA and OC (Figure S1), temporal analysis of the predicted OA/OC ratio at stations where only OC is available (Figure S2), boxplots showing the range of *R* ^2^ obtained by the six ML models at the different sites during LOSO cross-validation for HOA, BBOA, and OOA (Figure S3), time series of HOA, BBOA, and OOA at the Lille (France) station (Figure S4), feature importance of HOA, BBOA, and OOA using the CatBoost model (with log transformation) (Figure S5), residuals of HOA, BBOA, and OOA using the CatBoost model with log-transformed targets (Figure S6), scatter plots for HOA, BBOA, and OOA using the CAMx model vs PMF from AMS/ACSM (Figure S7), spatial and temporal analysis of OA fractions from CAMx (Figure S8), map showing coefficient of determination (*R* ^2^) values for HOA, BBOA, and OOA from LOSO cross-validation using the CatBoost model across all monitoring stations with the OA component data set across Europe (Figure S9), relative contribution of HOA, BBOA, and OOA to the total OA concentration for each site plotted in order of increasing latitude from 35 N to 70 N (Figure S10), scatter plots for HOA, BBOA, and OOA using the CatBoost model without OA as an input with log-transformed targets (Figure S11), positive matrix factorization (PMF): PMF of 44 AMS/ACSM data sets that resolved three primary organic aerosol components, OOA, HOA, and BBOA, with additional site-specific factors included in total OA but not modeled individually (section S.1), and data availability: mean concentrations for HOA, BBOA, and OOA for 180 sites (section S.2) (PDF)
