# Supplementary material for: Machine-Learning-Driven Reconstruction of Organic Aerosol Sources across Dense Monitoring Networks in Europe
Source: Environ Sci Technol Lett. 2025 Oct 20;12(11):1523–31. doi: 10.1021/acs.estlett.5c00771 (PMC12613817; doi:10.1021/acs.estlett.5c00771)
Supplement: Supplementary file 1 [file ez5c00771_si_001.pdf]

Supporting Information for publication: "Machine Learning-Driven Reconstruction of Organic Aerosol Sources Across Dense Monitoring Networks in Europe"

Adrien Jouanny[1], Abhishek Upadhyay*[1], Jianhui Jiang[2], Petros Vasilakos[1], Marta Via[3], Yun Cheng[4], Benjamin Flueckiger[5, 51], Gaëlle Uzu[6], Jean-Luc Jaffrezo[6], Céline Voiron[6], Olivier Favez[7], Hasna Chebaicheb[7, 8], Aude Bourin[8], Anna Font[8, 32], Véronique Riffault[8], Evelyn Freney[9], Nicolas Marchand[10], Benjamin Chazeau[10], Sébastien Conil[11], Jean-Eudes Petit[12], Jesús D. de la Rosa[13], Ana Sanchez de la Campa[13], Daniel Sanchez-Rodas Navarro[13], Sonia Castillo[14], Andrés Alastuey[15], Xavier Querol[15], Cristina Reche[15], María Cruz Minguillón[15], Marek Maasikmets[16], Hannes Keernik[16, 17], Fabio Giardi[18], Cristina Colombi[19], Eleonora Cuccia[19], Stefania Gilardoni[20], Matteo Rinaldi[21], Marco Paglione[21], Vanes Poluzzi[22], Dario Massabò[23], Claudio Belis[24], Stuart Grange[25, 26], Christoph Hueglin[26], Francesco Canonaco[27], Anna Tobler[27], Hilkka J. Timonen[28], Minna Aurela[28], Mikael Ehn[29], Iasonas Stavroulas[30], Aikaterini Bougiatioti[30], Konstantinos Eleftheriadis[31], Maria I. Gini[31], Olga Zografou[31], Manousos-Ioannis Manousakas[31], Gang Ian Chen[32], David Christopher Green[32, 33], Petra Pokorná[34], Petr Vodička[34], Radek Lhotka[34], Jaroslav Schwarz[34], Andrea Schemmel[35], Samira Atabakhsh[36], Hartmut Herrmann[36], Laurent Poulain[36], Harald Flentje[37], Liine Heikkinen[38], Varun Kumar[39], Hugo Anne Denier van der Gon[40], Wenche Aas[41], Stephen M. Platt[41], Karl Espen Yttri[41], Imre Salma[42], Anikó Vasanits[42], Benjamin Bergmans[43], Yulia Sosedova, Jaroslaw Necki[44], Jurgita Ovadnevaite[45], Chunshui Lin[46], Julija Pauraite[47], Michael Pikridas[48], Jean Sciare[48], Jeni Vasilescu[49], Livio Belegante[49], Célia Alves[50], Jay G. Slowik[1], Nicole Probst-Hensch[5, 51], Danielle Vienneau[5, 51], André S. H. Prévôt[1], Aniss Aiman Medbouhi[52], Daniel Trejo Banos[4], Kees de Hoogh[5, 51], Kaspar R. Daellenbach *[1], Ekaterina Krymova *[4], Imad El Haddad *[1,53]

1. PSI Center for Energy and Environmental Sciences, 5232 Villigen PSI, Switzerland
2. Global Institute for Urban and Regional Sustainability, School of Ecological and Environmental Sciences, East China Normal University, 200231 Shanghai, China.
3. Center for Atmospheric Research, University of Nova Gorica, SI-5000 Nova Gorica, Slovenia
4. Swiss Data Science Center, EPFL and ETH Zürich, 8092 Zürich, Switzerland
5. Swiss Tropical and Public Health Institute, Kreuzstrasse 2, 4123 Allschwil, Switzerland
6. University Grenoble Alpes, CNRS, IRD, INP-G, INRAE, IGE (UMR 5001), 38000 Grenoble, France
7. Institut National de l'Environnement Industriel et des Risques (INERIS), Verneuil-en-Halatte, 60550, France
8. IMT Nord Europe, Institut Mines-Télécom, Université de Lille, Centre for Energy and Environment, Lille, 59000, France
9. Laboratoire de Météorologie Physique, Université Clermont Auvergne-CNRS, Aubière, 63170, France
10. Aix Marseille Univ., CNRS, LCE, 13003 Marseille, France
11. Agence nationale pour la gestion des déchets radioactifs (ANDRA), RD960 55290 Bure, France
12. Laboratoire des Sciences du Climat et de l'Environnement (LSCE), Gif-sur-Yvette, 91190, France
13. CIQSO-Center for Research in Sustainable Chemistry, Associate Unit CSIC-University of Huelva "Atmospheric Pollution", Campus El Carmen s/n, 21071, Huelva, Spain
14. Department of Applied Physics, Universidad de Granada, Av. de la Fuente Nueva, 18071, Granada, Spain
15. Institute of Environmental Assessment and Water Research (IDAEA-CSIC), 08034 Barcelona, Spain
16. Air Quality and Climate Department, Estonian Environmental Research Centre (EERC), Marja 4D, 10617 Tallinn, Estonia
17. Now at Institute of Physics, University of Tartu, 50411 Tartu, Estonia.
18. National Institute for Nuclear Physics - Florence Section, via Sansone 1, 50019 Sesto Fiorentino (FI), Italy
19. Environmental Protection Agency of Lombardy (ARPA Lombardia), 20124 Milan, Italy
20. Italian National Research Council, Institute of Polar Sciences (CNR-ISP), Milan, 20125, Italy
21. Italian National Research Council – Institute of Atmospheric Sciences and Climate (CNR-ISAC), Bologna, 40129, Italy
22. Arpae Emilia-Romagna, Centro Tematico Regionale Qualità dell'Aria, Bologna, 40139, Italy
23. Department of Physics University of Genoa & INFN-Genoa, Liguria, 16146, Italy
24. European Commission, Joint Research Centre, Ispra, 21027, Italy
25. School of Earth and Atmospheric Sciences, Queensland University of Technology, Gardens Point, Brisbane, Queensland 4000, Australia
26. Empa, Swiss Federal Laboratories for Materials Science and Technology, 8600 Dübendorf, Switzerland

27. Datalystica Ltd., Villigen, 5234, Switzerland
28. Atmospheric Composition Research, Finnish Meteorological Institute, Helsinki, 00560, Finland; Aerosol Physics Laboratory, Tampere University, Tampere, 33100, Finland
29. Institute for Atmospheric and Earth System Research/Physics, Faculty of Science, University of Helsinki, Helsinki, 00014, Finland
30. Institute for Environmental Research and Sustainable Development, National Observatory of Athens, Athens, 15236, Greece
31. Environmental Radioactivity & Aerosol Technology for Atmospheric & Climate Impact Lab, INRaSTES, NCSR "Demokritos", Athens 15310, Greece
32. MRC Centre for Environment and Health, Environmental Research Group, Imperial College London, 86 Wood Lane, London, W12 0BZ, United Kingdom
33. HPRU in Environmental Exposures and Health, Imperial College London, 86 Wood Lane, London, W12 0BZ, United Kingdom
34. Institute of Chemical Process Fundamentals, Czech Academy of Sciences, Prague 6, 165 00, Czech Republic
35. Umweltbundesamt, 06844 Dessau-Roßlau, Germany
36. Leibniz Institute for Tropospheric Research (TROPOS), Leipzig, 04318, Germany
37. German Meteorological Service (DWD), Hohenpeissenberg, 82383, Germany
38. Department of Environmental Science, Stockholm University, Stockholm, 114 18, Sweden; Bolin Centre for Climate Research, Stockholm University, Stockholm, 114 18, Sweden
39. Department of Environmental Sciences, Aarhus University, DK-4000, Denmark
40. TNO, Department of Climate, Air and Sustainability, 3584 CB Utrecht, Netherlands
41. NILU, Kjeller, 2007, Norway
42. Institute of Chemistry, Eötvös Loránd University, 1053 Budapest, Hungary
43. ISSEP – Institut Scientifique de Service Public, 4000 Liège, Belgium
44. AGH University of Krakow, Faculty of Physics and Applied Computer Science, Krakow, 30-059, Poland
45. School of Natural Sciences, Physics, University of Galway, Centre for Climate and Air Pollution Studies, Ryan Institute, Galway, H91 CF50, Ireland
46. State Key Laboratory of Loess Sciences, Center for Excellence in Quaternary Science and Global Change, Institute of Earth Environment, Chinese Academy of Sciences, 710061, Xi'an, China
47. SRI Center for Physical Sciences and Technology (FTMC), Vilnius, 10257, Lithuania
48. Climate and Atmosphere Research Center (CARE-C), The Cyprus Institute, Nicosia, 2121, Cyprus
49. National Institute of Research and Development for Optoelectronics INOE2000, Magurele, 077125, Romania
50. Department of Environment and Planning, CESAM – Centre for Environmental and Marine Studies, University of Aveiro, 3810-193, Aveiro, Portugal
51. University of Basel, 4001 Basel, Switzerland
52. Department of Intelligent Systems, KTH Royal Institute of Technology, 11428 Stockholm, Sweden
53. College of Environmental Sciences and Engineering, Peking University, 100084 Beijing, China

* Imad El-Haddad: imad.el-haddad@psi.ch
* Abhishek Upadhyay: abhishek.upadhyay@psi.ch
* Ekaterina Krymova: ekaterina.krymova@sdsc.ethz.ch
* Kaspar R. Daellenbach: kaspar.daellenbach@psi.ch

Table S1: List of the 44 stations that have OA, HOA, BBOA, and OOA measurements, with available the number of daily concentrations(n), time range for data availability and references describing measurement and PMF methodology. Stations highlighted in gray are rural or suburban stations, while the others are urban stations.

Location	n	From	To	Reference
Athens DEM	n=329	2017-11	2018-10	1
Athens NOA	n=317	2016-07	2017-08	1
Barcelona	n=310	2017-09	2018-10	1
Basel	n=91	2013-01	2013-12	2

Bern	n=90	2013-01	2013-12	[2]
Birkenes	n=700	2016-01	2018-09	[1]
Bologna	n=183	2011-11	2014-06	[3]
Bucharest	n=353	2016-09	2017-08	[1]
Carnsore Point	n=347	2016-08	2017-08	[1]
Creil Faiencerie	n=943	2016-01	2019-11	[4], (Hasna Chebaicheb, under preparation)
Dublin	n=358	2016-09	2017-08	[1]
Finokalia	n=22	2011-09	2011-10	[5]
Frauenfeld	n=90	2013-01	2013-12	[2]
Gennevilliers	n=456	2018-01	2019-09	[4], (Hasna Chebaicheb, under preparation)
Helsinki	n=336	2017-06	2018-05	[1]
Hohenpeissenberg	n=901	2017-01	2019-12	[1]
Hyytiälä	n=402	2017-07	2018-10	[1]
Košetice	n=229	2019-01	2019-10	[1]
Kraków	n=377	2018-01	2019-04	[1]
Lille	n=821	2016-10	2019-12	[1,6]
London	n=698	2015-11	2018-02	[1]
Lyon Centre	n=1552	2015-03	2019-11	[4], (Hasna Chebaicheb, under preparation)
Magadino	n=91	2013-01	2013-12	[2]
Magadino	n=414	2013-08	2014-10	[1]
Marseille	n=617	2011-02	2019-12	[1,4], (Hasna Chebaicheb, under preparation)
Melpitz	n=330	2016-09	2017-08	[1]
Metz Borny	n=450	2018-03	2019-10	[4], (Hasna Chebaicheb, under preparation)
Montsec	n=66	2011-07	2011-09	[2]
Mt. Cimone (CMN)	n=25	2012-06	2012-07	[3]
Paris Les Halles	n=87	2019-10	2019-12	[4], (Hasna Chebaicheb, under preparation)

Paris SIRTA	n=1606	2011-10	2019-12	[1,4], (Hasna Chebaicheb, under preparation)
Payerne	n=91	2013-01	2013-12	[2]
Poitiers Augouard	n=1272	2015-11	2019-12	[4], (Hasna Chebaicheb, under preparation)
Puy de Dôme	n=242	2015-04	2016-02	[1]
San Pietro Capofiume	n=93	2011-11	2013-10	[3]
San Vittore	n=90	2013-01	2013-12	[2]
SMEARII	n=38	2011-03	2011-04	[5]
St Gallen	n=91	2013-01	2013-12	[2]
Strasbourg Danube	n=32	2019-11	2019-12	[4], (Hasna Chebaicheb, under preparation)
Talence Bordeaux	n=1031	2016-01	2019-12	[4], (Hasna Chebaicheb, under preparation)
Tartu	n=291	2016-09	2017-07	[1]
Vaduz	n=91	2013-01	2013-12	[2]
Zurich	n=390	2011-02	2013-12	[2]
Zurich	n=265	2016-08	2017-07	[1]

Table S2: 136 stations that have OC measurements, n is the number of daily concentrations. Stations highlighted in gray are rural or suburban stations, while the others are urban stations. (*): OA measurements were available and imputed directly in the model.

**Location**	**PM2.5/PM10**	**From**	**To**	**Location**	**PM2.5/PM10**	**From**	**To**
AGH University Krakow	0/69	2018-02	2018-10	Aix-en-provence	0/117	2013-07	2014-07
Alfragide	29/0	2014-02	2015-10	Aosta-Saint Christophe	27/0	2018-01	2018-03
Aspvreten	0/591	2010-01	2014-12	Athens	120/3	2013-02	2014-02
Bailen	10/535	2010-01	2019-10	Barcelona	51/669	2013-01	2019-12
Basel-Binningen	88/0	2018-06	2019-05	Bassi	119/12	2013-01	2014-02
Bern-Bollwerk	84/4	2018-06	2019-05	Birkenes	186/324	2010-01	2019-12

Brockau	0/58	2019-01	2019-12	Budapest center	0/42	2014-02	2018-07
Burg	0/732	2010-01	2015-12	Cabauw Wielsekade	363/511	2012-01	2019-12
Cabauw Zijdeweg	91/0	2011-01	2011-12	Campisabalos	247/45	2010-01	2016-12
Campus Univ El Carmen	0/608	2010-02	2019-12	Capannori	0/157	2019-03	2019-12
Carranque	0/255	2010-01	2014-12	Chamonix	0/120	2013-11	2014-10
Chemnitz-Leipziger Str.	0/60	2019-01	2019-12	Coimbra	0/100	2018-12	2019-06
Coimbra	0/99	2018-12	2019-06	Collmberg	0/598	2010-01	2019-12
Copenhagen_HCAB	0/1731	2015-01	2019-12	Copenhagen_HCØ	0/724	2018-01	2019-12
Copenhagen_Hvidovre	0/690	2018-01	2019-12	DEM_Athens	331/0	2017-01	2019-12
Diabla Gora	3165/0	2010-01	2019-12	Domäne Bobbe	0/708	2010-01	2015-12
Donon	128/0	2018-02	2019-12	Dresden-Bergstr.	0/605	2010-01	2019-12
Els Torms	56/0	2016-01	2016-12	Finokalia	0/1024	2013-01	2018-03
GRE-fr	0/253	2013-01	2018-03	Genoa Bolzaneto	117/0	2011-05	2011-10
Granada	0/102	2017-12	2019-09	Granada Norte	0/329	2010-01	2015-12
Grande Synthe	0/113	2018-01	2019-12	Guipry	342/0	2014-01	2019-11
Görlitz	0/58	2019-01	2019-12	Halle_Merseburger Strasse	0/681	2010-01	2015-12
Halle_Nord	0/465	2012-01	2015-12	Halle_Paracelsusstr.	0/575	2011-01	2015-12
Harwell	0/628	2010-01	2011-12	Hurdal	115/350	2011-01	2019-12
Hyytiälä	0/14	2017-12	2018-01	Illmitz	29/0	2017-11	2018-03
Iskrba	2085/0	2010-01	2019-12	Ispra	3370/0	2010-01	2019-12
Jove-Gijón	0/73	2019-05	2019-12	K-puszta	0/71	2010-01	2018-04
Kosetice (NAOK)	2092/0	2010-01	2019-12	Krakow	0/63	2018-01	2018-09
Kårvatn	121/341	2011-01	2019-12	La Línea	0/264	2010-01	2014-12
La Rabida	0/308	2015-01	2019-12	Leipzig Lützner Str.	0/605	2010-01	2019-12

Leipzig-Mitte	0/604	2010-01	2019-12	Leipzig-West	0/604	2010-01	2019-12
Lens	0/285	2011-03	2014-05	Lepanto	0/277	2010-01	2014-12
Lyon	0/122	2012-01	2012-12	MONTALE	0/218	2013-11	2015-01
MRS-5av	0/512	2011-07	2017-12	Madrid Ensanche vallecas	0/30	2019-10	2019-12
Madrid Escuelas Aguirre	0/60	2019-07	2019-12	Magadino-Cadenazzo	87/0	2018-06	2019-05
Magdeburg_Reuterallee	0/661	2010-01	2015-12	Magdeburg_West	0/482	2012-01	2015-12
Manlleu	116/90	2013-11	2017-01	Marczell Gyorgy	0/42	2017-10	2018-04
Matalascañas	0/495	2010-01	2019-05	Matorova	28/1375	2014-02	2019-12
Mediterráneo	0/279	2010-01	2014-12	Melpitz	722/2894	2010-01	2019-12
Milano Pascal	162/2623	2012-01	2019-12	Milano Senato	0/869	2017-01	2019-12
MilanoShivenoglia	225/629	2017-01	2019-12	Moguer	0/253	2010-01	2014-12
Monte Martano	0/337	2018-01	2019-12	Monte Velho	0/60	2014-02	2015-12
Montsec	0/249	2016-01	2019-12	Montseny	283/787	2010-01	2019-12
Namur	0/101	2014-06	2015-10	Neuglobsow	606/0	2010-06	2019-12
Nice	0/228	2014-06	2016-12	Niesky	0/59	2019-01	2019-12
Nogent	0/226	2013-01	2018-05	Norunda Stenen	0/213	2018-01	2019-12
Observatoire Perenne de l'Environnement	466/487	2011-11	2019-12	Oulanka	107/0	2012-01	2012-05
Paris SIRTA	815/0	2017-01	2019-12	Payerne	432/1	2010-01	2019-12
Peyrusse Vieille	326/0	2013-01	2019-12	Pic du Midi	0/131	2017-01	2019-12
Plza Castillo	0/267	2010-01	2014-12	Poitiers	0/120	2014-12	2016-01
Port de Bouc	0/307	2014-06	2018-11	Porto	110/9	2013-01	2014-01
Puente Mayorga	0/270	2010-01	2014-12	Puy de Dôme	475/0	2010-01	2014-12
Revin	547/168	2011-01	2019-12	Rigi	287/0	2010-01	2019-12

Roubaix	0/159	2013-01	2014-05	Rouen	0/168	2013-01	2014-06
Saint-Nazaire-le-Désert	358/0	2014-01	2019-12	San Fernando	0/258	2010-01	2014-12
San Pablo de los Montes	259/0	2012-03	2016-12	Schauinsland	583/0	2010-06	2019-12
Schmücke	590/0	2010-06	2019-12	Schwartenberg	0/58	2019-01	2019-12
Sealand_Risø	0/1711	2015-01	2019-12	Sonnblick	0/29	2017-10	2018-04
Strasbourg	0/80	2013-04	2014-04	Talence	0/160	2012-02	2013-04
Tartu	0/17	2017-12	2018-03	Turin	0/124	2013-07	2014-07
Utö	137/2680	2011-07	2019-12	Vavihill	0/624	2010-01	2015-12
Verneuil	354/0	2014-01	2019-12	Vielsalm	0/166	2014-05	2018-04
Villanueva del Arzobispo	128/2	2014-06	2015-06	Virolahti	190/2812	2010-09	2019-12
Víznar	60/0	2016-01	2016-12	Waldhof	660/0	2010-06	2019-12
Wittenberg_Dessauer Strasse	0/729	2010-01	2015-12	Zarra	56/0	2016-02	2016-12
Zeppelin Mountain, Svalbard	0/99	2017-01	2019-12	Zielonka	699/0	2016-01	2019-12
Zingst	190/0	2017-01	2019-12	Zürich-Kaserne	88/0	2018-06	2019-05
Prague - Suchdol	308* (PM1)	2012-06	2013-05	Mace Head	196* (PM1)	2011-02	2011-10

Table S3: Variables used as input features for HOA, BBOA, and OOA modelling.

Group	Variable(s)	Buffers (m)	Source	Description
Time	Year	-	-	-
	Month	-	-	-
	Day of week	-	-	-
Position	Latitude	-	-	-
	Longitude	-	-	-
	OA mass concentration	-	-	-
Road Variables	road_class_1_x	200, 500, 1000, 2000	Open street maps	Total length of major roads within the buffer
	road_class_2_x	200, 500, 1000, 2000		Total length of big roads within the buffer

	road_class_3_x	200, 500, 1000, 2000		Total length of small roads within the buffer
Land Use Variables	Corine Land Cover classification	-	CORINE Cover version 20 (Copernicus, 2021a)[7], Copernicus services (Copernicus, 2021b)[8]	-
	Agriculture, Airports, Barren, IMD, Natural Green, Ports, Roads & Railways, Transport, Urban Fabric, Urban Green, Water, Wetlands	500, 1000		Various land-use classifications
	Population Density	500, 1000	GEOSTAT population grid (European Comission, 2011)[9]	-
WRF variables	2-meter temperature	-	-	-
	Relative humidity	-	-	-
	Surface atmospheric pressure	-	-	-
	Surface wind speed and direction	-	-	-
	Planetary boundary layer height	-	-	-
CAMx variables	NO3, SO4, NH4, O3, NO, NO2, CO, BBOA, HOA, OOA	-	-	Concentrations

As model inputs we have used HOA, BBOA and OOA simulated using a chemical transport model (CAMx). These variables are not colinear as they arise from different sources and atmospheric processes. To generate these fractions, CAMx simulates PM and VOC emissions from different sectors, their chemical transformation (e.g. reactions with OH and O3), their atmospheric transport and deposition. Therefore, we have not found the need to use emissions and chemical variables as inputs to the ML model, as this would result in some redundancy and deteriorate the performance of the model.

We have used in addition total OA estimated based on OC measurements as model input. This has significantly improved the performance of the ML model. In Figure S11 we present the scatter plots without the use of total OA as inputs.

Table S4: Description of used Machine learning and Deep learning models.

	Details	Model	Parameters
Ensemble learning	Log normalization of targets. Raw inputs.	Random forest[10]	n_estimators=200, max_depth=24, max_features=0.467, min_samples_leaf=10, min_samples_split=6, criterion='squared_error',

			random_state=42
		LightGBM[11]	random_state=42,<br>force_col_wise=True,<br>objective='poisson',<br>bagging_fraction=0.6067,<br>bagging_freq=10,<br>feature_fraction=0.757,<br>lambda_l1=2.441,<br>lambda_l2=70.742,<br>learning_rate=0.0907,<br>max_depth=14,<br>min_data_in_leaf=50,<br>num_leaves=255
		CatBoost[12]	random_seed=42,<br>loss_function='RMSE',<br>bagging_temperature=0.8188,<br>border_count=237,<br>depth=9,<br>iterations=1988,<br>l2_leaf_reg=2.6611,<br>learning_rate=0.0192,<br>one_hot_max_size=50,<br>random_strength=1.926
Deep learning	Softmax layer. The target was ($\frac{HOA}{OA}$,$\frac{BBOA}{OA}$, $\frac{OOA}{OA}$,$\frac{res_{OA}}{OA}$). Features were normalized.	Multi-Layer Perceptron[13,14]	hidden_layers = [32, 32, 32],<br>lr = 1e-2,<br>num_epochs = 600,<br>batch_size = 512,<br>criterion = L1Loss,<br>activation = Tanh,<br>dropout_prob = 0.3,<br>patience_early_stop = 40,<br>lr_scheduler = ReduceLROnPlateau,<br>lr_step = 15,<br>lr_var = 0.5
		β-Variational Auto-Encoder[15,16]	latent_size = 32,<br>hidden_size_1 = 128,<br>hidden_size_2 = 64,<br>dropout_rate = 0.01,<br>epochs = 100,<br>batch_size = 512,<br>beta = 2,<br>lr = 1e-4,<br>activation = LeakyReLU<br>lr_decay = 1e-5,<br>gamma = 0.99,

Table S5: Variables used as features OA/OC ratio modelling.

Group	Variable(s)	Buffers (m)	Source	Description
Time	Year	-	-	-
	Month	-	-	-
	Day of week	-	-	-
Position	Latitude	-	-	-
	Longitude	-	-	-
Road Variables	road_class_1_x	200, 500, 1000, 2000	Open street map	Total length of major roads within the buffer
	road_class_2_x	200, 500, 1000, 2000		Total length of big roads within the buffer
	road_class_3_x	200, 500, 1000, 2000		Total length of small roads within the buffer
Land Use Variables	Corine Land Cover classification	-	CORINE Cover version 20 (Copernicus, 2021a)[7], Copernicus services (Copernicus, 2021b)[8]	-
	Agriculture, Airports, Barren, IMD, Natural Green, Ports, Roads & Railways, Transport, Urban Fabric, Urban Green, Water, Wetlands	500, 1000		Various land-use classifications
	Population Density	500, 1000	GEOSTAT population grid (European Comission, 2011)[9]	-
WRF and CAMx Variables	2-meter temperature	-	-	-
	Relative humidity	-	-	-
	Surface atmospheric pressure	-	-	-
	Surface wind speed and direction	-	-	-
	Planetary boundary layer height	-	-	-
	NO3, SO4, NH4, O3, NO, NO2, CO, BBOA, HOA, OOA	-	-	Concentrations
CAMx derived variables	HOC	$= 0.82 * HOA_{camx}$		
	BBOC	$= 0.62 * BBOA_{camx}$		
	OOC	$= 0.55 * OOA_{camx}$		
	OA_{camx}	$= HOA_{camx} + BBOA_{camx} + OOA_{camx}$		
	OC_{camx}	$= HOC_{camx} + BBOC_{camx} + OOC_{camx}$		
	OA/OC ratio	$= \frac{OA_{camx}}{OC_{camx}}$		

As model inputs we have used HOA, BBOA and OOA simulated using a chemical transport model (CAMx). These variables are not colinear as they arise from different sources and atmospheric processes. To generate these fractions, CAMx simulates PM and VOC emissions

from different sectors, their chemical transformation (e.g. reactions with OH and O3), their atmospheric transport and deposition. Therefore, we have not found the need to use emissions and chemical variables as inputs to the ML model, as this would result in some redundancy and deteriorate the performance of the model.

HOC, BBOC, OOC were computed for training, as follows: $\begin{cases} HOC_{PMF} = 0.82 * HOA_{PMF} \\ BBOC_{PMF} = 0.62 * BBOA_{PMF} \\ OOC_{PMF} = 0.55 * OOA_{PMF} \end{cases}$.

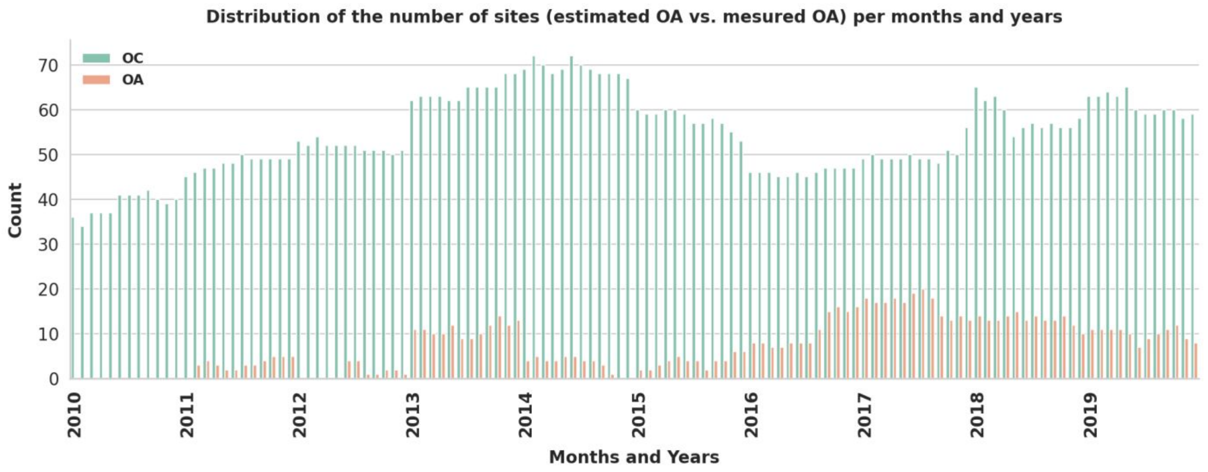

Figure S1: Distribution of the number of sites per year and per month with OA (orange) and with OC (blue).

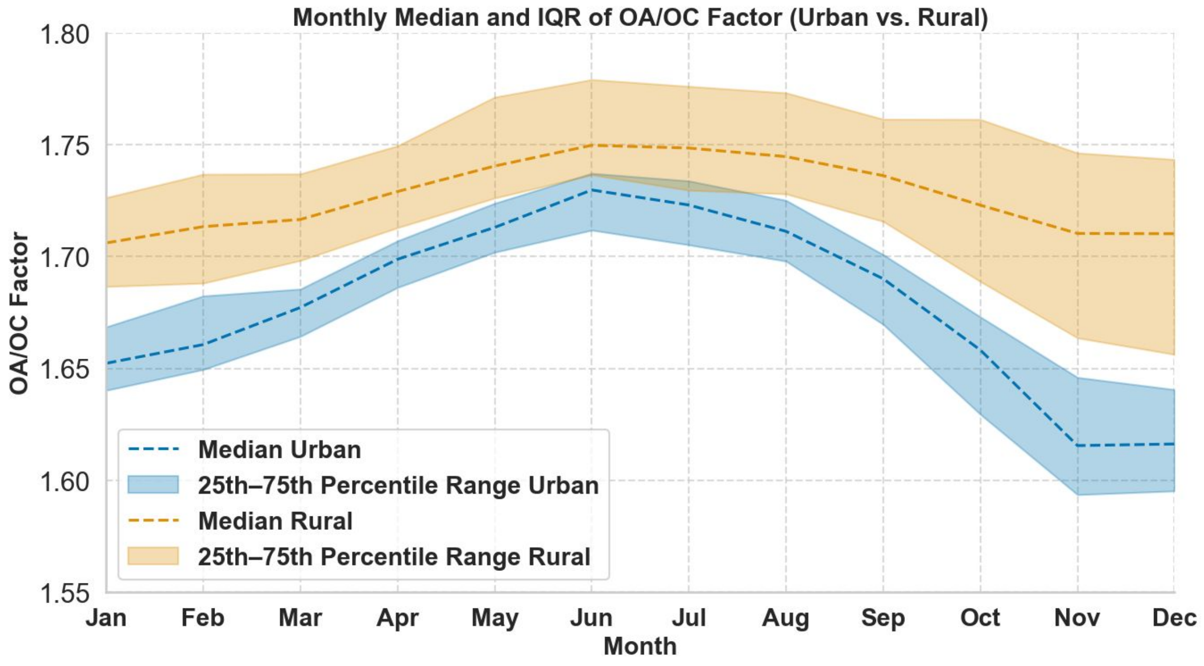

Figure S2: Temporal analysis of the predicted OA/OC ratio at stations where only OC is available. We differentiated urban and rural areas using land use variables that correspond to population density and imperviousness density (IMD) described in Table S3.

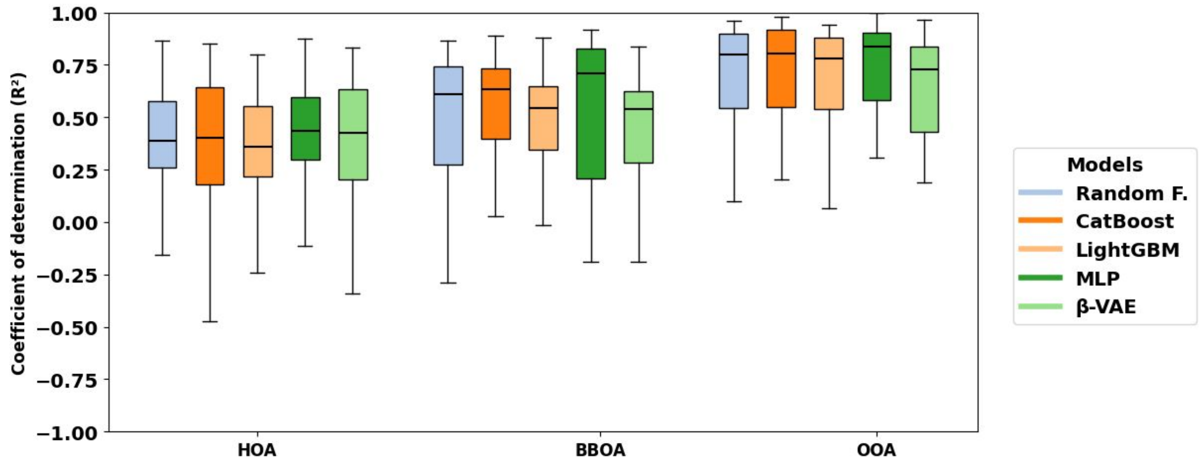

Figure S3: Boxplots showing the range of R^2 obtained by the six ML models at the different sites during leave-one-site-out (LOSO) cross-validation for HOA, BBOA and OOA. R^2 was computed as follows $R^2 = 1 - \frac{\|y_{true} - y_{pred}\|_2^2}{\|y_{true} - y_{mean}\|_2^2}$.

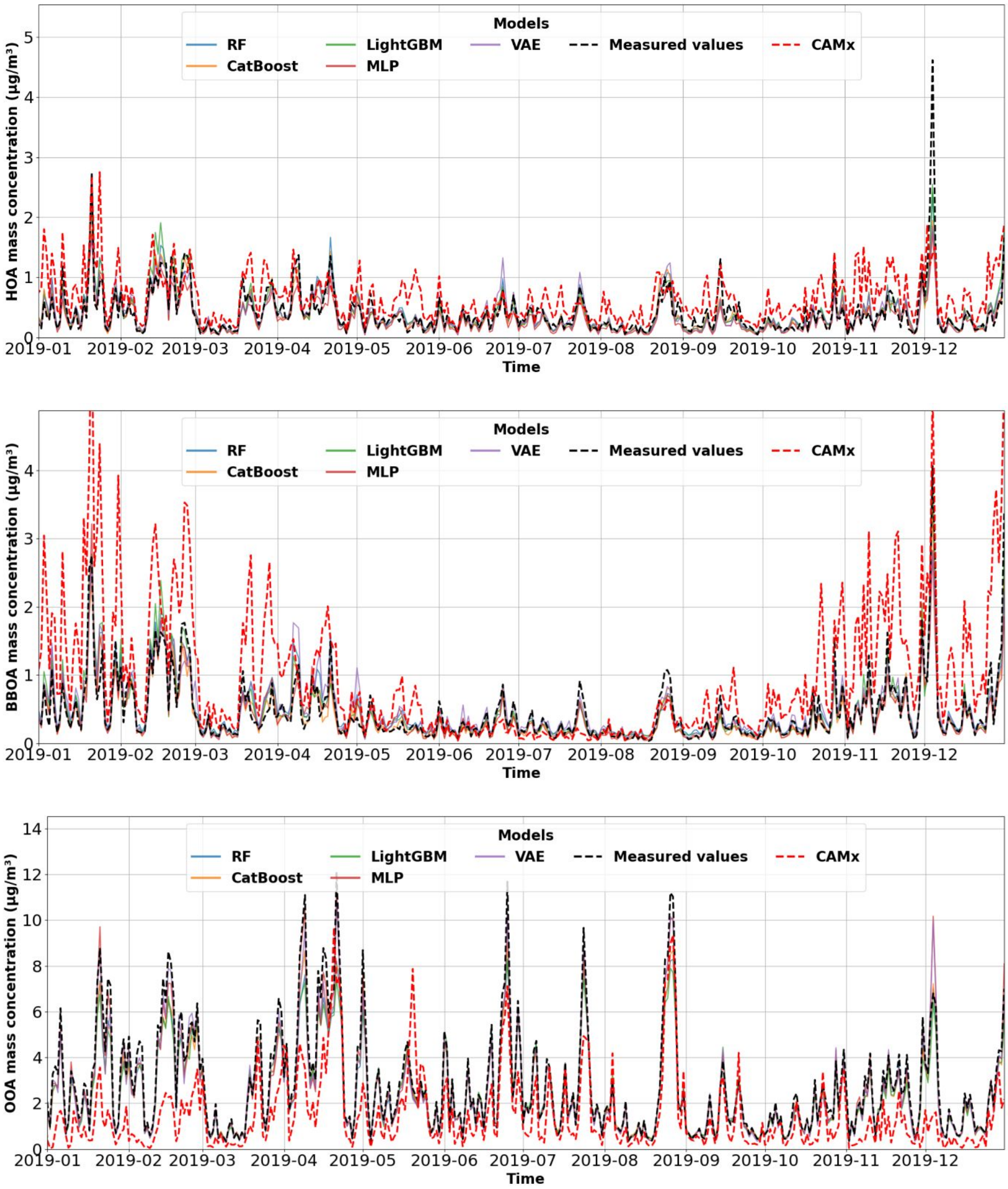

Figure S4: Time series of HOA, BBOA and OOA at Lille (France) station.

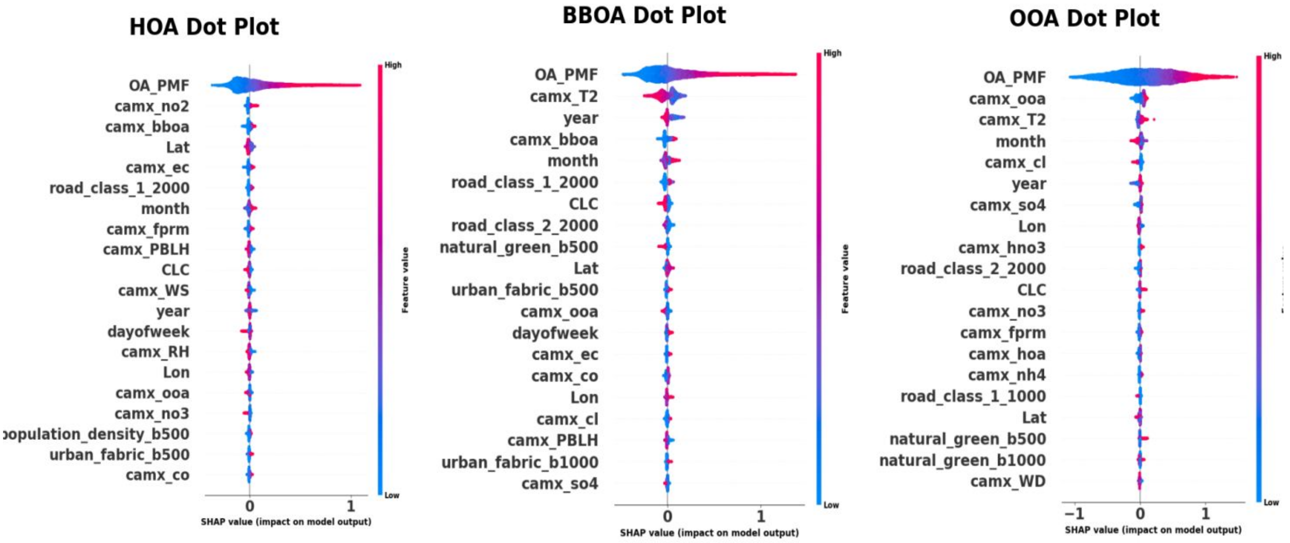

Figure S5: Feature importance of HOA, BBOA and OOA using the CatBoost model (with log-transformation). These SHAP plots show how each feature contributes to increasing or decreasing the predicted value for a given model. Each point represents a single data point, positioned according to the feature's "SHAP value", which quantifies that feature's effect on shifting the model's prediction away from an average baseline.

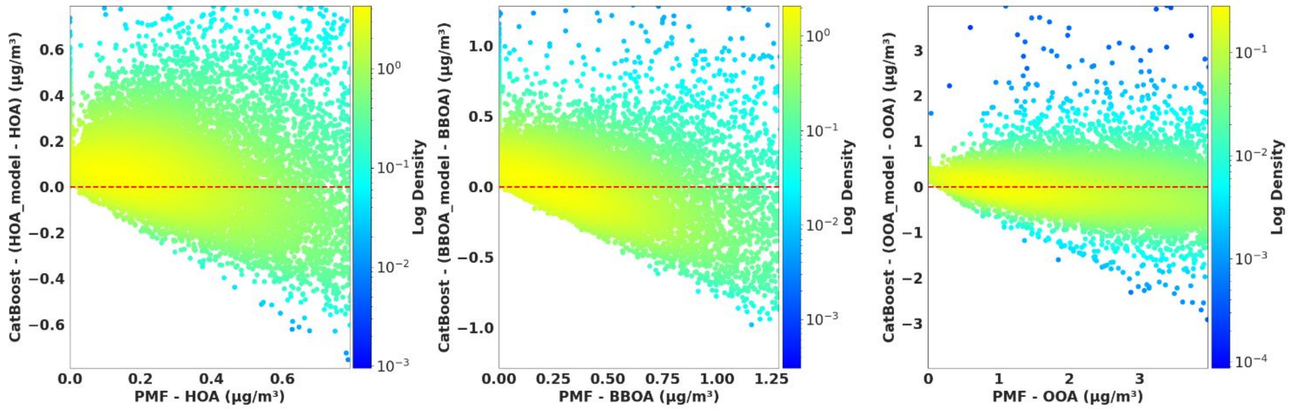

Figure S6: Residuals of HOA, BBOA and OOA using Catboost model with log-transformed targets. Residuals are defined as the following $Res_{HOA} = HOA_{PMF} - HOA_{Catboost}$.

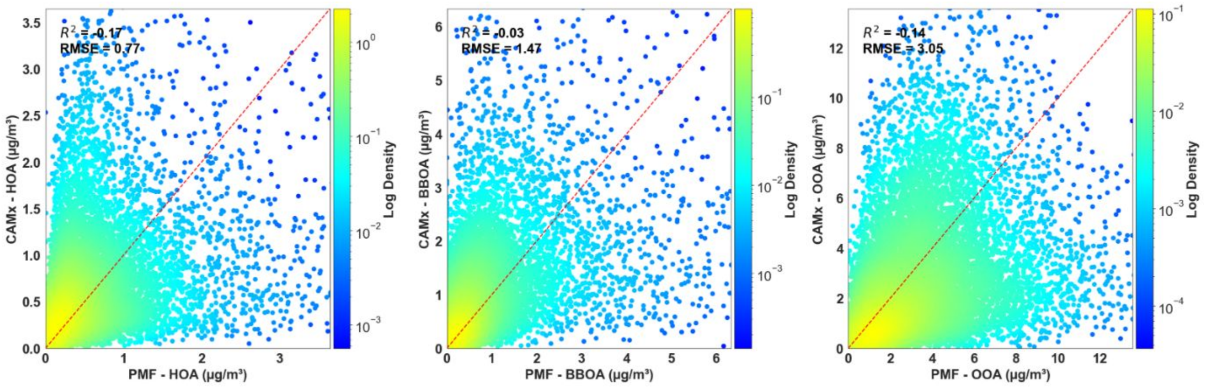

Figure S7: Scatter plots for HOA, BBOA and OOA using the CAMx model vs. PMF from AMS/ACSM. The red line corresponds to y=x.

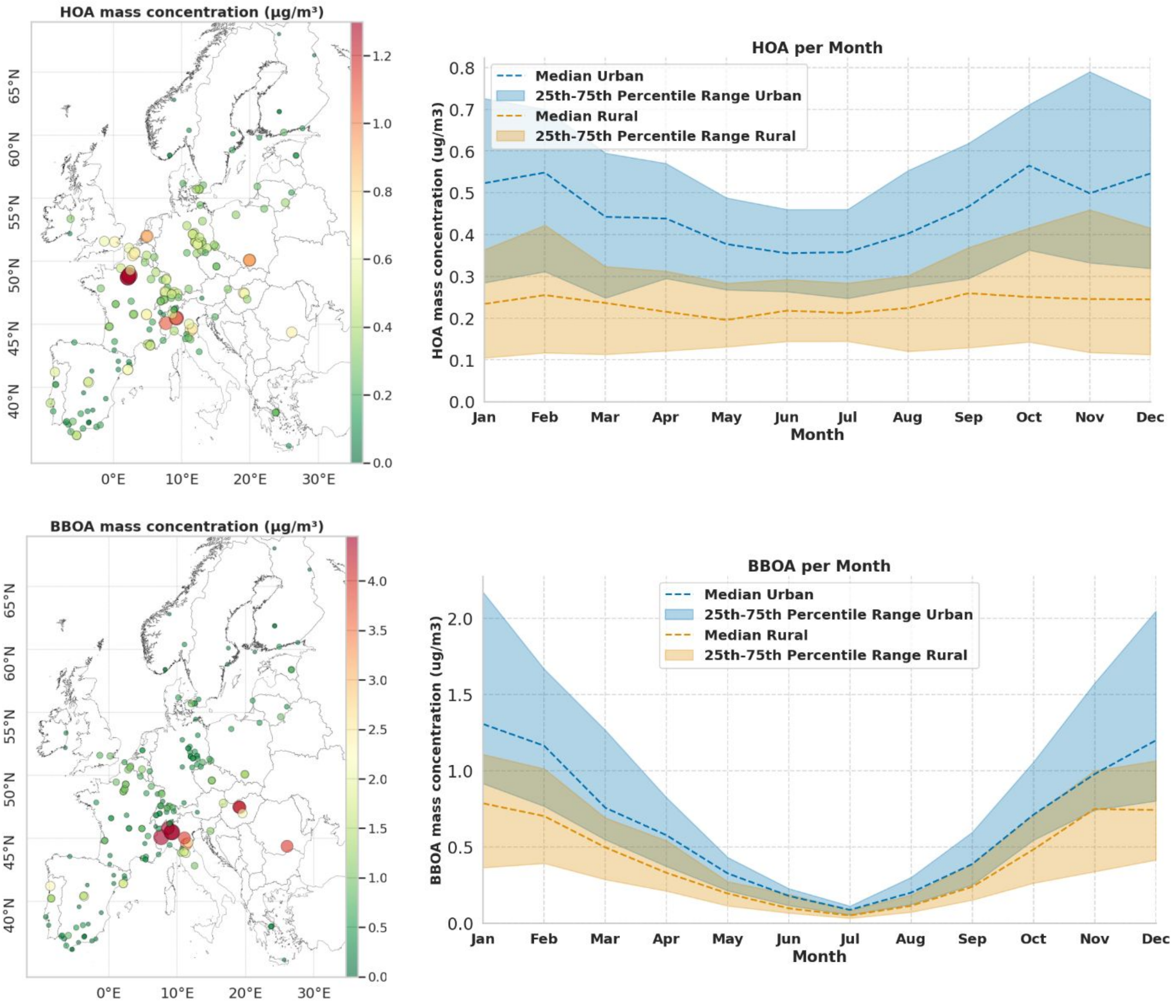

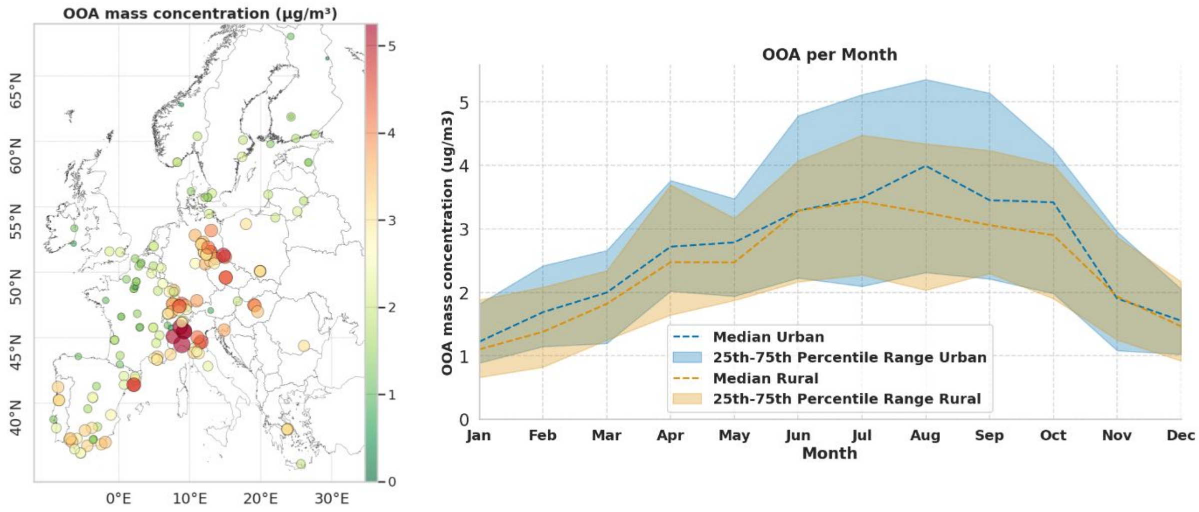

Figure S8: Spatial and temporal analysis of OA fractions from CAMx. We differentiated urban and rural areas using land use variables that correspond to population density and imperviousness density (IMD) described in Table S3.

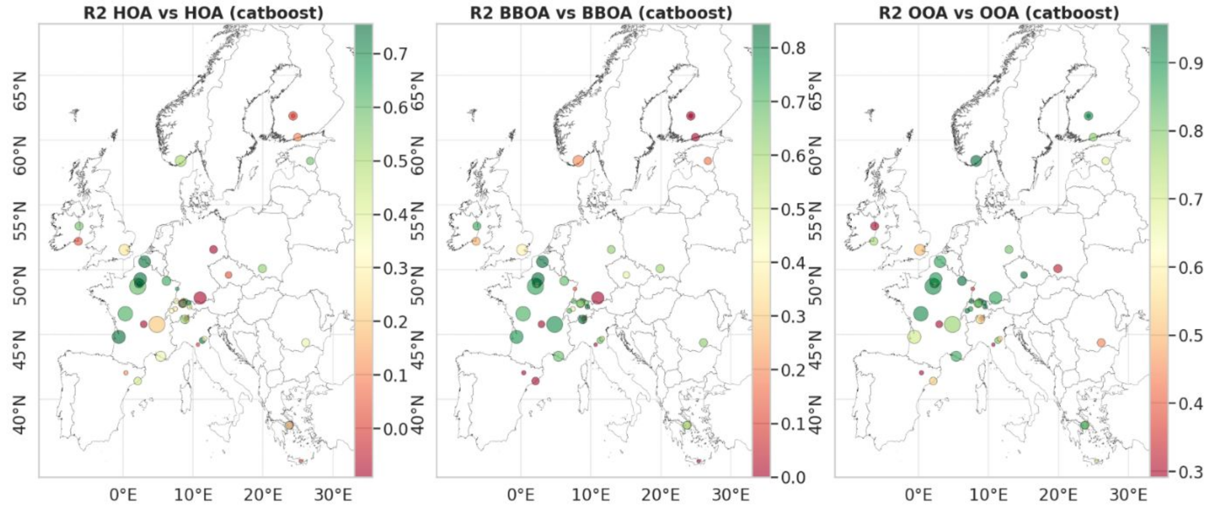

Figure S9: Map showing coefficient of determination (R^2) values for HOA, BBOA, and OOA from leave-one-site-out (LOSO) cross-validation using the CatBoost model across all monitoring stations with OA component dataset across Europe.

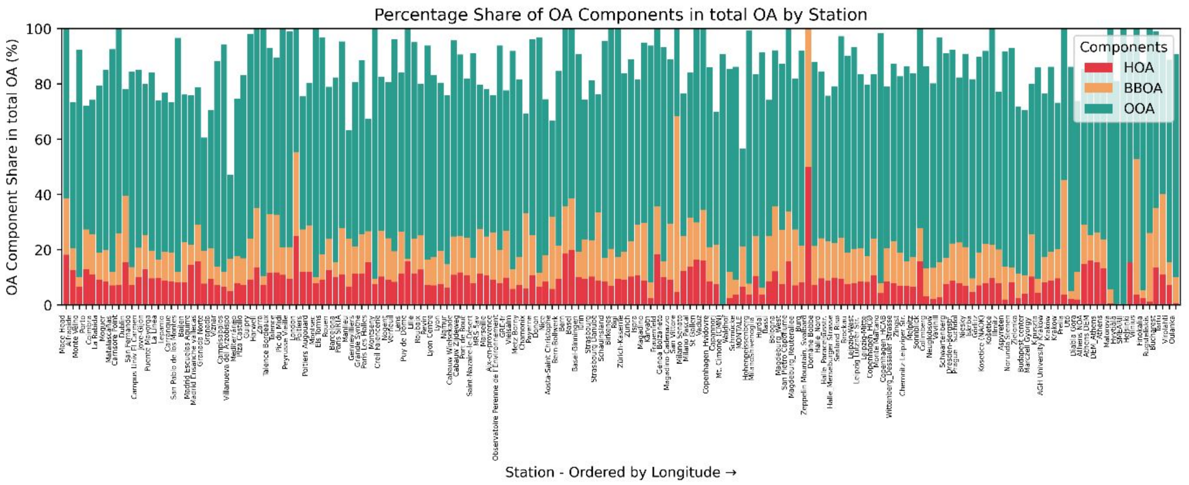

Figure S10: Relative contribution of HOA, BBOA and OOA to the total OA concentration for each site plotted in order of increasing latitude from 35N to 70N.

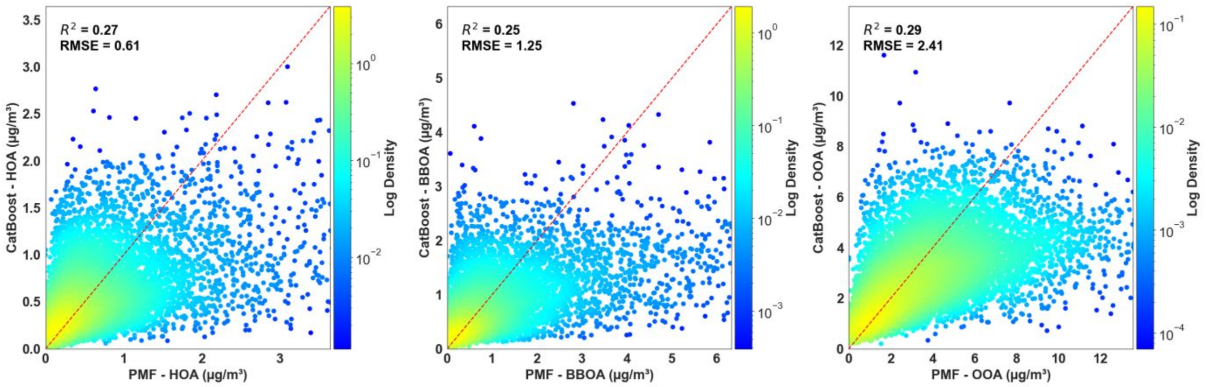

Figure S11: Scatter plots for HOA, BBOA and OOA using the CatBoost model without OA as an input with log-transformed targets, points colored according to the log density of the best two-dimensional Gaussian.

S.1 Positive matrix factorization (PMF)

The organic aerosol (OA) components used in this study were derived from 44 ground-based measurement campaigns using Aerosol Chemical Speciation Monitors (ACSM) and Aerosol Mass Spectrometers (AMS). Source apportionment was conducted using Positive Matrix Factorization (PMF), a statistical technique introduced by Paatero and Tapper[17] (1994) that resolves the OA mass spectral variability into a linear combination of static factor profiles and their time-dependent contributions.

All studies broadly followed the harmonized PMF approach described in Crippa et al.[18] (2014), which provides detailed guidance on factor extraction and identification in AMS/ACSM datasets. Table S1 lists the references which provide the methodological details for each dataset used in this analysis.

The number of factors retained in each PMF solution was chosen based on residual analysis and the interpretability of the extracted factors. Factor identification was performed using established criteria, including:

- **Mass spectral profiles:** Diagnostic ions such as m/z 43 ($C_2H_3O^+$), m/z 44 (CO_2^+), m/z 57 ($C_4H_9^+$), and m/z 60 ($C_2H_4O_2^+$).
- **Temporal behavior:** Diurnal patterns indicative of emission sources or atmospheric processes.
- **Correlation with external tracers:** Such as levoglucosan, elemental carbon (EC), black carbon (BC), and inorganic secondary aerosol components (NH_4^+, NO_3^-, SO_4^{2-}).

Across the 44 datasets, three factors were most commonly and consistently identified:

- **OOA (Oxygenated Organic Aerosol):** Characterized by dominant signals at m/z 44 (CO_2^+) and m/z 43 ($C_2H_3O^+$), low diurnal variability, and high correlation with secondary inorganic species. In many cases, OOA was further resolved into two sub-fractions—less and more oxygenated types—based on the relative dominance of m/z 43 vs. 44. These subtypes were summed up in our analysis to represent total OOA.
- **HOA (Hydrocarbon-like Organic Aerosol):** Identified by strong signals at m/z 57 and 59, a clear traffic-related diurnal pattern (e.g., morning and evening peaks), and correlations with EC or traffic-related BC.
- **BBOA (Biomass Burning Organic Aerosol):** Recognized by elevated m/z 60 and 73, representing anhydrous sugar fragments, nighttime increases, and correlations with levoglucosan (when available) or solid fuel BC.

At a limited number of sites, additional factors were resolved, including:

- **COA (Cooking Organic Aerosol):** Typically peaking during meal times and exhibiting strong m/z 55 and 57 signals.
- **Site-specific sources:** Linked to local emissions.

While these additional factors were included in the total OA input for model training, they were not modeled individually as outputs due to their limited occurrence across sites.

Only a few studies conducted formal uncertainty assessments, typically using bootstrapping techniques. Reported uncertainties ranged from 10–25% for OOA and 20–40% for HOA and BBOA. Although uncertainty estimates were not available for every dataset, the use of a harmonized PMF approach enhances the inter-site comparability of OA components and supports the robustness of these data as training labels in our machine learning models.

S.2 Data availability

Data, including the mean concentrations for HOA, BBOA, and OOA for 180 sites are available at the following link https://drive.switch.ch/index.php/s/By88RFK0Q2NgyWw.

References:

1. Chen G, Canonaco F, Tobler A, Aas W, Alastuey A, Allan J, et al. European aerosol phenomenology − 8: Harmonised source apportionment of organic aerosol using 22 Year-long ACSM/AMS datasets. Environ Int. 2022 Aug;166:107325.

2. Daellenbach KR, Stefenelli G, Bozzetti C, Vlachou A, Fermo P, Gonzalez R, et al. Long-term chemical analysis and organic aerosol source apportionment at nine sites in central Europe: source identification and uncertainty assessment. Atmospheric Chem Phys. 2017;17(21):13265–82.

3. Paglione M, Gilardoni S, Rinaldi M, Decesari S, Zanca N, Sandrini S, et al. The impact of biomass burning and aqueous-phase processing on air quality: a multi-year source apportionment study in the Po Valley, Italy. Atmospheric Chem Phys. 2020 Feb 3;20(3):1233–54.

4. Chebaicheb H, de Brito JF, Amodeo T, Couvidat F, Petit JE, Tison E, et al. Multiyear high-temporal-resolution measurements of submicron aerosols at 13 French urban sites: data processing and chemical composition. Earth Syst Sci Data. 2024;16(11):5089–109.

5. Daellenbach KR, Uzu G, Jiang J, Cassagnes LE, Leni Z, Vlachou A, et al. Sources of particulate-matter air pollution and its oxidative potential in Europe. Nature. 2020 Nov 1;587(7834):414–9.

6. Chebaicheb H, Brito JF de, Chen G, Tison E, Marchand C, Prévôt ASH, et al. Investigation of four-year chemical composition and organic aerosol sources of submicron particles at the ATOLL site in northern France. Environ Pollut. 2023;330:121805.

7. CORINE Cover version 20 (Copernicus, 2021a). Available from: https://land.copernicus.eu/en/products/corine-land-cover (accessed 2024-12-13).

8. Copernicus services (Copernicus, 2021b). Available from: https://land.copernicus.eu/en/products/high-resolution-layer-imperviousness (accessed 2024-12-13).

9. GEOSTAT population grid (European Comission, 2011). Available from: https://www.eea.europa.eu/data-and-maps/data/external/geostat-2011-grid-dataset (accessed 2024-12-13).

10. Breiman L. Random forests. Mach Learn. 2001;45(1):5–32.

11. Ke G, Meng Q, Finley T, Wang T, Chen W, Ma W, et al. LightGBM: a highly efficient gradient boosting decision tree. In: Proceedings of the 31st International Conference on Neural Information Processing Systems. Red Hook, NY, USA: Curran Associates Inc.; 2017. p. 3149–57. (NIPS'17).

12. Prokhorenkova L, Gusev G, Vorobev A, Dorogush AV, Gulin A. CatBoost: unbiased boosting with categorical features. In: Proceedings of the 32nd International Conference on Neural Information Processing Systems. Red Hook, NY, USA: Curran Associates Inc.; 2018. p. 6639–49. (NIPS'18).

13. Gardner MW, Dorling SR. Artificial neural networks (the multilayer perceptron)—a review of applications in the atmospheric sciences. Atmos Environ. 1998 Aug;32(14–15):2627–36.

14. Murtagh F. Multilayer perceptrons for classification and regression. Neurocomputing. 1991 July;2(5–6):183–97.

15. Higgins I, Matthey L, Pal A, Burgess C, Glorot X, Botvinick M, et al. beta-VAE: Learning Basic Visual Concepts with a Constrained Variational Framework. Available from: https://openreview.net/forum?id=Sy2fzU9gl (accessed 2024-12-13).

16. Roskams-Hieter B, Wells J, Wade S. Leveraging Variational Autoencoders for Multiple Data Imputation. In: Koutra D, Plant C, Gomez Rodriguez M, Baralis E, Bonchi F, editors. Machine Learning and Knowledge Discovery in Databases: Research Track. Cham: Springer Nature Switzerland; 2023. p. 491–506.

17. Paatero P, Tapper U. Positive matrix factorization: A non-negative factor model with optimal utilization of error estimates of data values. Environmetrics. 1994;5(2):111–26.

18. Crippa M, Canonaco F, Lanz VA, Äijälä M, Allan JD, Carbone S, et al. Organic aerosol components derived from 25 AMS data sets across Europe using a consistent ME-2 based source apportionment approach. Atmospheric Chem Phys. 2014;14(12):6159–76.
